# Systematic screening with information and home sampling for genital Chlamydia trachomatis infections in young men and women in Norway: a randomized controlled trial

**DOI:** 10.1186/1471-2334-13-30

**Published:** 2013-01-23

**Authors:** Hilde Kløvstad, Olav Natås, Aage Tverdal, Preben Aavitsland

**Affiliations:** 1 Norwegian Institute of Public Health, PO box 4404, Nydalen, Oslo, 0403, Norway; 2 Stavanger University Hospital, PO box. 8100, Forus, Stavanger, 4068, Norway; 3 Current adress: Epidemi, Lasarettet, Kristiansand, 4610, Norway

## Abstract

**Background:**

As most genital *Chlamydia trachomatis* infections are asymptomatic, many patients do not seek health care for testing. Infections remain undiagnosed and untreated. We studied whether screening with information and home sampling resulted in more young people getting tested, diagnosed and treated for chlamydia in the three months following the intervention compared to the current strategy of testing in the health care system.

**Method:**

We conducted a population based randomized controlled trial among all persons aged 18–25 years in one Norwegian county (41 519 persons). 10 000 persons (intervention) received an invitation by mail with chlamydia information and a mail-back urine sampling kit. 31 519 persons received no intervention and continued with usual care (control). All samples from both groups were analysed in the same laboratory. Information on treatment was obtained from the Norwegian Prescription Database (NorPD). We estimated risk ratios and risk differences of being tested, diagnosed and treated in the intervention group compared to the control group.

**Results:**

In the intervention group 16.5% got tested and in the control group 3.4%, risk ratio 4.9 (95% CI 4.5-5.2). The intervention led to 2.6 (95% CI 2.0-3.4) times as many individuals being diagnosed and 2.5 (95% CI 1.9-3.4) times as many individuals receiving treatment for chlamydia compared to no intervention in the three months following the intervention.

**Conclusion:**

In Norway, systematic screening with information and home sampling results in more young people being tested, diagnosed and treated for chlamydia in the three months following the intervention than the current strategy of testing in the health care system. However, the study has not established that the intervention will reduce the chlamydia prevalence or the risk of complications from chlamydia.

**Trial registration:**

ClinicalTrials.gov IDNCT00283127

## Background

Genital Chlamydia trachomatis infection may, if left untreated, lead to infertility, ectopic pregnancy, neonatal infection and facilitation of HIV infection [1-4]. It is the most commonly reported sexually transmitted infection in Europe [5], and in Norway the yearly number of diagnosed cases has been increasing over the last few years. In 2011, 22 530 cases were diagnosed; 68% were below 25 years of age of whom 67% were female [6,7].

Early diagnosis and treatment has been considered a major strategy to prevent complications and further transmission of the infection [8,9] although the evidence for this effect on a population level is weak [10]. In Norway, testing for genital C. trachomatis is done in the health care system through testing of symptomatic persons, partner tracing or through opportunistic screening of men and women below 25 years of age [11]. Testing is widely available. In 2011, the testing rate in Norway was 5468/100 000 population [6]. Still, as most infections are asymptomatic [12,13] many patients do not seek health care for testing. Therefore opportunistic screening or even systematic screening programmes have been advocated. However, there is a rising concern that opportunistic as well as systematic screening of young individuals for C. trachomatis may not be effective control measures [14].

The objective of this study was to determine whether systematic screening with information and home sampling would result in more men and women aged 18–25 years being tested, diagnosed and treated for genital C. trachomatis in the three months following the interventions compared to the current strategy of testing in the health care system in the same period. (Genital C. trachomatis infection will throughout the article be referred to as chlamydia).

## Methods

**Figure 1:**
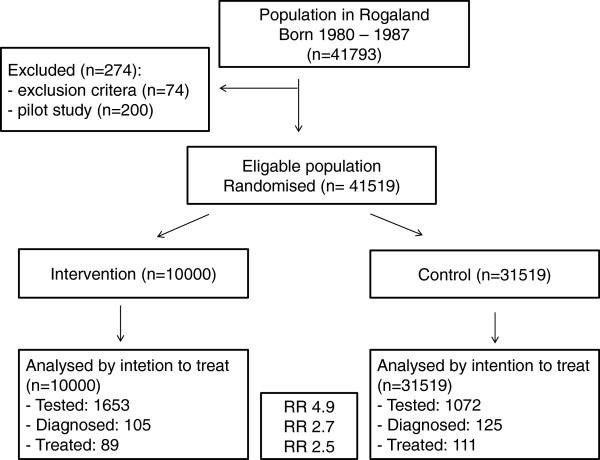
Flow chart of randomized trial of home sampling as an intervention to test, diagnose and treat persons aged 18–25 years for genital Chlamydia trachomatis infection, Rogaland county, Norway 2006.

We conducted a population based parallel randomized controlled trial (RCT) among all persons aged 18–25 years (birth years 1980–1987) registered in the national population register in one Norwegian county, Rogaland. On November 11 2005, this population was 41 793 persons (Figure 1). We excluded those who did not have a postal address (74) or had been invited to our pilot study (200). The study period lasted four months, February-May in 2006.

### Intervention

Persons assigned to the intervention group (10 000) received a mail package at their home address consisting of the following: a letter with information on chlamydia and the importance of testing and treatment and an invitation to take a home test free of charge, a urine container, a durable water-tight plastic container, instructions on how to obtain a first void urine sample, a prepaid return envelope and a questionnaire (socio-demographic details, sexual behaviour, symptoms (discharge, endocervical bleeding, pelvic pain, urethral itching, dysuria) and history of sexually transmitted infections (STI)). Participants were asked to mail the urine samples by post in a leak-proof vessel enclosed in a durable water-tight plastic container directly to the laboratory at Stavanger University Hospital within three months after receiving the invitation. We used no reminders. In order to avoid overload in the laboratory by all participants mailing their urine samples at the same time, the intervention group was divided into four subgroups according to municipality of residence. The subgroups received the invitation one week apart and were then observed for the next three months. (Schedule: 30 January −30 April, 6 February- 6 May, 13 February −13 May, 20 February −20 May).

A letter containing the test result and a contact phone number for support was provided to all participants from the diagnosing laboratory. If the test result was positive, the participant was requested to visit their family general practitioner, another doctor or a youth clinic for treatment and partner tracing at no cost.

### Current strategy

Persons assigned to the control group (31 519) received no intervention and were not informed about the trial and thus continued with the current strategy of testing in the health care system, including clinically indicated testing, partner tracing and opportunistic screening. Samples obtained in the health care system included either cervical or urethral swabs or first void urine samples. Patients with positive test results were, as per current routines, contacted by health professional for treatment and partner tracing. Test and treatment were free of charge. The control group was also followed for three months divided into four subgroups according to municipality and corresponding with the intervention group; starting and ending the observation period on the same dates as the corresponding intervention subgroup.

### Definitions and data collection

#### Testing

A participant was defined as having been tested if at least one urine sample, cervical or urethral swab for chlamydia testing had been submitted to Stavanger University Hospital (the laboratory serving Rogaland county with chlamydia testing) within the study period. Information was obtained from the laboratory database and added to the study dataset.

#### Diagnosis

A participant was defined as having been diagnosed with chlamydia if at least one test, obtained at home or in the health care system, was positive for chlamydia within the study period. Information was obtained from the laboratory database and added to the study dataset.

#### Treatment

A participant was defined as having been treated for chlamydia if the person had filled at least one prescription for a drug against chlamydia (azitromycin, doksycyklin, erytromycin, lymecyklin, amoxicillin) within 30 days following a positive test result. Information was obtained from the Norwegian Prescription Database (NorPD) which contains information from all filled prescriptions in Norway from 2004. The study dataset was encrypted, made pseudonymous (by a third party) and merged with NorPD. The pseudonyms were removed before our analysis and no personal identifiers were available for the researchers.

### Outcome measures and statistics

The primary outcome measures were the risk ratio of being tested, diagnosed and treated defined as the proportion (risk) tested/diagnosed/treated in the intervention group (10 000) divided by the proportion (risk) tested/diagnosed/treated in the control group (31 519). Secondary outcome measure were the risk ratio of being treated given a positive test defined as the proportion treated among the diagnosed in the intervention group divided by the proportion treated among the diagnosed in the control group. We also compared the two groups by the risk difference.

We also present risk ratios of being tested, diagnosed and treated stratified by age group and gender and the prevalence of infection by gender and age groups in each of the study groups.

We calculated 95% confidence intervals for all risk ratios and risk differences. We applied intention-to-treat-analysis which is an analysis based on the initial group assignment (Figure 1). This is done to avoid that various reasons for not participating in the assigned group will interfere with the randomization and introduce bias. For our primary outcome measures the denominator in the intervention group is therefore 10 000 and in the control group 31 519. We used Poisson regression as the exponential of the regression coefficient for the intervention (yes-no) variable has the interpretation of a risk ratio. Interaction was assessed in the Poisson models with and without the interaction term, and based on the likelihood ratio test. We used Stata (StataCorp 2005. Stata Statistical Software: Release 9. College Station, TX: StataCorp LP) for analysis.

### Diagnostic method

All samples were analyzed with BD ProbeTec Chlamydia Amplified DNA assay (Becton, Dickinson, Franklin Lake, New Jersey, USA). A sensitivity of > 90% and a specificity of > 99% have been reported for this test, but with a somewhat lower sensitivity when testing urine from women [15,16]. DNA was extracted from urine using the BUGS’n BEADS™ STI preparation system (Genpoint, Oslo, Norway) [17]. Amplification controls were not used.

### Home samples obtained from participants living outside the county

In the analysis, we excluded test and treatment information from persons in the intervention group who returned a home sample but stated that they lived outside Rogaland county (for instance students). This was done in order to ensure an equal representation of participants who were in the population register but living outside the county in both the intervention and control group. While mailed invitations for home testing may have been forwarded to the temporary address of the participants in the intervention group, patients tested and diagnosed in health care facilities outside the county could not be assessed because their samples were analysed in other laboratories.

### Randomization

The participants were randomized into one of two parallel groups.

*Sequence generation:* We used the unique personal identity number of individuals living in Norway to randomize participants. Personal identity numbers is generated by the national population register which is part of The Norwegian Tax Administration*.* The national population register includes all persons who live (or have lived) in Norway and registers information on births, deaths, addresses, immigration and emigration. The 11 digit personal identity number contains the following: The first six digits represent birth date (ddmmyy). The next three digits are given consecutively from 000–499 to every new citizen in Norway born (or immigrated) to Norway the last half of the 20^th^ century. The last of these three digits indicates the persons’ gender. The last two digits are control numbers estimated after modulus 11 based on the previous 9 and 10 numbers [18]. Modulus eleven is mathematical algorithm were each of the previous digits in the personal id number is multiplied by its weight, the results of the multiplication are added together, this product is divided by the modulus number 11, the remainder is subtracted from the modulus number 11 giving the control number.

*Mechanism used to implement the allocation sequence:* The eligible population was sorted in ascending order according to these two last digits of their personal identity number. The first 10 000 participants was selected for the intervention group and the remaining 31 519 for the control group (allocation ratio 1:3.15). *Implementation:* The allocation sequence was implemented by a third party (contracted from the population register). The list of assigned individuals was provided to the investigators by this third party. Participants (in the intervention group) were enrolled by an invitation letter sent by the Norwegian Institute of Public Health to the participants’ home addresses. *Allocation concealment mechanism:* Individuals in the intervention group were aware of the study and their group assignment. The control group received no information about the study. Laboratory personnel were aware that mailed urine containers came from participants in the intervention group.

### Sample size calculation

We calculated sample size with the Cohort Power module of the Episheet calculator for all three primary outcomes, but used results for the least frequent one (risk ratio of being treated) to guide the study’s sample size. We first assumed that 16.7% of the control group members would get tested in a three-month period, based on earlier published testing rates for women aged 20–24 in Norway [19,20]. We then assumed that 8% of those tested would have a positive test, based on national surveillance data [6]. Finally, we assumed that 90% of those with positive tests would get treated. The risk for this outcome in the control group was then 0.167 × 0.08 × 0.9 = 0.012. With a maximum of 10 000 individuals in the intervention group (for economical reasons) we found that we would need at least 26 000 individuals in the control group to achieve a power of 90% to detect a risk ratio of 1.5 or higher, with an alpha level of 0.05.

### Non - responders

To determine factors influencing the response to the home sampling intervention, a random selection of 3000 non–responders (did not submit urine sample or return the questionnaire within the defined study period) was sent the same questionnaire - but not the home sampling kit - once more with additional questions on reason why they chose not to participate.

### Ethics

Informed consent was not obtained from the participants in either of the assigned groups. The trial was approved by the Regional Committee for Medical and Health Research Ethics (REK), South –East Region and was registered at ClinicalTrials.gov (ClinicalTrials.gov ID NCT00283127) in January 2006.

## Results

The two groups had the same age and sex distribution. Municipality of residence was unevenly distributed in the two groups (Table 1). Adjustments for municipality had minimal effect on the risk ratio estimates. Therefore, only unadjusted results are shown. Of all the invitations, 429 (4.3%) were returned unopened due to the wrong address. All were included in the analysis.

**Table 1:** Baseline characteristics intervention group (n=10 000) and control group (n= 31 519), Rogaland county, Norway 2006

	**Intervention group n= 10 000**		**Control group n= 31 519**	
	**n**	**(%)**	**n**	**(%)**
**Age (18–21)**	5005	50.1%	15889	50.4%
**Sex (male)**	5077	50.7%	16002	50.7%
**Municipality:**				
Bjerkheim	71	0.7%	213	0.7%
Bokn	12	0.1%	51	0.2%
Eigersund	340	3.4%	1068	3.4%
Finnøy	72	0.7%	1068	0.7%
Forsand	32	0.3%	69	0.2%
Gjesdal	250	2.5%	808	2.6%
Hå	428	4.3%	1299	4.1%
Haugesund	826	8.3%	2549	8.1%
Hjelmeland	61	0.6%	216	0.7%
Karmøy	883	8.8%	2984	9.5%
Klepp	383	3.8%	1031	3.3%
Kvitsøy	8	0.1%	42	0.1%
Ølen	97	1.0%	263	0.8%
Lund	92	0.9%	263	0.8%
Randaberg	219	2.2%	725	2.3%
Rennesøy	90	0.9%	231	0.7%
Sandnes	1588	15.9%	4859	15.4%
Sauda	132	1.3%	362	1.1%
Sokndal	68	0.7%	270	0.9%
Sola	489	4.9%	1554	4.9%
Strand	276	2.8%	872	2.8%
Suldal	103	1.0%	307	1.0%
Time	400	4.0%	1253	4.0%
Tysvær	209	2.1%	735	2.3%
Utsira	9	0.1%	10	0.0%
Vindafjord	137	1.4%	379	1.2%

After three months, 16.5% of the population in the intervention group had been tested for chlamydia at least once whereas in the control group 3.4% had been tested (Table 2), risk ratio 4.9 (95% CI 4.5-5.2). The majority of those tested in the intervention group (87%, 1433/1653) got tested during the first half of the three months. In the control group, date of testing was evenly distributed throughout the period. The intervention increased the probability of being diagnosed with chlamydia by 2.6 times (95% CI 2.0 -3.4) and for being treated for chlamydia by 2.5 times (95% CI 1.9-3.4). There was no difference between the groups in the probability of being treated given a positive test (RR 0.95, 95% CI 0.86-1.1).

**Table 2:** Risk of being tested, diagnosed and treated for genital Chlamydia trachomatis in the intervention and control groups, the risk ratios and the risk differences in a randomized trial of information and home sampling, Rogaland county, Norway 2006

**Outcome**	**Intervention group**		**Control group**		**Risk ratio (95% confidence interval)**	**Risk difference (95% confidence interval)**
	**n/N**	**Risk (%)**	**n/N**	**Risk (%)**		
**Tested**	1653/10000	16.5	1072/31519	3.4	4.9 (4.5 – 5.2)	13.1% (12.4 – 13.9)
**Tested and diagnosed**	105/10000	1.05	125/31519	0.40	2.6 (2.0 – 3.4)	0.65% (0.44 – 0.86)
**Tested, diagnosed and treated**	89/10000	0.89	111/31519	0.35	2.5 (1.9 – 3.4)	0.54% (0.34 –0.73)

The proportion of persons tested in the intervention group was higher in women than in men; women 20% (980/4923), men 13% (673/5077). According to protocol we also performed the analyses by gender and age group. The effect of the intervention varied by age (p interaction = 0.001) and by sex (p interaction =0.000) with (proportion) tested as outcome. The effect of the intervention was higher among men (RR 9.3, 95% CI 8.0-10.8) than women (RR 3.7, 95% CI 3.4-4.0) for the outcome proportion tested (Table 3). With proportion diagnosed and treated as outcomes there was no interaction (p varied between 0.15 and 0.89). In the intervention group, 41% (673/1653) of the tested were male compared to only 21% (229/1072) in the control group.

**Table 3:** Risk of being tested, diagnosed and treated for genital Chlamydia trachomatis in the intervention and control groups, the risk ratios and the risk differences by age group and gender in a randomized trial of information and home sampling, Rogaland county, Norway 2006

**Outcome**		**Intervention**		**Control**		**Risk ratio (95% confidence interval)**	**Risk difference (95% confidence interval)**
		**n/N**	**Risk (%)**	**n/N**	**Risk (%)**		
**Women 18–21 year**	Tested	507/2453	20.6	485/7829	6.2	3.3 (3.0 – 3.7)	14.5% (12.8 – 16.2)
	Diagnosed	37/2453	1.5	63/7829	0.8	1.9 (1.3 – 2.8)	0.70% (0.18 – 1.2)
	Treated	34/2453	1.4	58/7829	0.7	1.9 (1.2 – 2.9)	0.65% (0.15 – 1.2)
**Women 22**–**25 year**	Tested	473/2470	19.1	358/7687	4.7	4.1 (3.6 – 4.7)	14.5% (12.9 – 16.1)
	Diagnosed	29/2470	1.2	18/7687	0.2	5.0 (2.8 – 9.0)	0.94% (0.50 – 1.4)
	Treated	26/2470	1.1	16/7687	0.2	5.1 (2.7 – 9.4)	0.84% (0.43 – 1.3)
**All women**	Tested	980/4923	19.6	843/15516	2.3	3.7 (3.4 – 4.0)	14.5% (13.3 – 15.6)
	Diagnosed	66/4923	1.3	81/15516	0.5	2.6 (1.9 – 3.5)	0.82% (0.48 – 1.2)
	Treated	60/4923	1.2	74/15516	0.5	2.6 (1.8 – 3.6)	0.74% (0.42 – 1.1)
**Men 18**–**21 year**	Tested	322/2552	12.6	124/8060	1.5	8.2 (6.7 – 10.0)	11.1% (9.8 – 12.4)
	Diganosed	20/2552	0.8	26/8060	0.3	2.4 (1.4 – 4.3)	0.46% (0.10 – 0.82)
	Treated	15/2552	0.6	23/8060	0.3	2.1 (1.1 – 3.9)	0.30% (0 – 0.62)
**Men 22**–**25 year**	Tested	351/2525	13.9	105/7942	1.3	10.5 (8.5 – 13.0)	12.6% (11.2 – 14.0)
	Diagnosed	19/2525	0.8	18/7942	0.2	3.3 (1.7 – 6.3)	0.53% (0.17 – 0.88)
	Treated	14/2525	0.6	14/7942	0.2	3.1 (1.5 – 5.6)	0.38% (0.07 – 0.68)
**All men**	Tested	673/5077	12.4	229/16002	1.4	9.3 (8.0 – 10.8)	11.8% (10.9 – 12.8)
	Diagnosed	39/5077	0.8	44/16002	0.3	2.8 (1.8 – 4.3)	0.49% (0.24 – 0.75)
	Treated	29/5077	0.6	37/16002	0.2	2.5 (1.5 – 4.0)	0.34% (0.12 – 0.56)

Among those tested, the proportion chlamydia positive in the intervention group was 6.3% (6.7% in females, 5.8% in males) and in the control group 11.9% (9.6% in females, 19.2% in males) (Table 4). The proportion of those diagnosed who received treatment was 85% (85/105) in the intervention group and 89% (111/125) in the control group. Less men than women received treatment for chlamydia. In the intervention group 74% (23/29) of the men filled a prescription for chlamydia vs 91% (60/66) among women. In the control group the corresponding figures were 84% (37/44) vs 91% (74/81). Of the infections detected in the intervention group 70% (74/105) were asymptomatic, 70% (46/66) in women and 72% (28/39) in men.

**Table 4:** Proportion positive for genital Chlamydia trachomatis infection by age group and gender among those tested in the randomized trial, Rogaland county, Norway 2006

**Group**	**Intervention group**		**Control group**	
	**Diagnosed/tested**	**% positive (95% confidence interval)**	**Diagnosed/tested**	**% positive (95% confidence interval)**
Men 18–21 years	20/322	6.2 (3.9-9.2)	26/124	21.0 (14.5-28.9)
Men 22–25 years	19/351	5.4 (3.4-8.2)	18/105	17.1 (10.8-25.2)
Women 18–21 years	37/507	7.2 (5.3-9.8)	63/485	12.9 (10.2-16.2)
Women 22–25 years	29/473	6.1 (4.2-8.6)	18/358	5.0 (3.1-7.7)

Among the tests in the intervention group, 85% were home tests and the remaining 15% were tests from the health care system of which the proportion positive test results were 16% (40/252). 2.6% (22 persons) of the chlamydia tests in the control group were home tests sent to participants in the intervention group, but used by participants in the control group (forwarded by those who received the invitation).

### Non-responders

Only 9.2% (277/3000) replied to the questionnaires for non-responders. Main reasons for non-participation were “I don’t believe I am infected” (53%), “I forgot to take the urine test” (32%) and “I have recently been tested at the doctor’s office” (25%). 19% reported that they had never had sexual intercourse. Due to the poor response rate, we were unable to determine factors influencing the acceptability of home sampling.

## Discussion

In this population-based randomized controlled trial we found that home sampling led to the identification and treatment of 2.5 times more individuals infected with chlamydia than the current strategy of testing in the health care system in the same three months period. This is the first randomized trial to demonstrate how such an intervention influences the number of people treated for chlamydia.

### Comparison with other trials

The total response to the invitation for home sampling in this study was low compared to population based home sampling trials conducted in other countries. In Denmark, the proportion tested was 39% for women and 27% for men [21]. In the Netherlands the proportion tested was 41% in a pilot chlamydia population study [22] whereas in the first screening round of a comprehensive register based chlamydia screening implementation programme the proportion tested was 16% [23]. In the UK the proportion tested was 31.5% [24]. All studies showed a lower uptake in young adults, and in males as shown in our study. In contrast, an RCT conducted among US males showed an uptake of only 7.8% [25].

We did not send a reminder. This may have contributed to a lower response rate, given that 32% of the non-responders reported they had forgotten to take the test. A positive effect of reminders on the uptake has, however, not been universal [24,26,27]. The level of testing in regular care would influence the additional benefit of offering home sampling in a population. Comparable testing rates between countries are difficult to obtain. Although Norway is likely to have high testing rates on a European level [28,29] we cannot conclude that this fully explains the relatively low uptake of testing in our study.

Other randomized trials of screening with home sampling have, like this study, demonstrated increased testing [30]. Andersen et al. found a relative risk of being tested of 4.1 (95% CI 3.8-4.4) for women and 19.1 for men (95% CI 16.0-22.8) in Denmark [21]. Scholes et al. found a relative risk of 11.1 among U.S males [25]. In contrast to these studies, we were able to demonstrate also an effect on treatment of diagnosed infections. We found that the intervention overall had less impact on this outcome measure, probably because it preferentially increased the testing of individuals with lower risk of infection than those who seek the health care system for chlamydia testing at their own initiative. This effect was especially seen among men since the men in the control group were less likely to get tested unless they had a high risk of infection. Data from the national surveillance supports this finding. The positivity rate is higher among men than women. In 2011, the positivity rate in the age group 15–19 was 15.6% for men and 13.1% for women. In the age group 20–24 it was 16.7% for men and 10.2% for women [6].

The proportion chlamydia positive in the intervention group is in the upper range of what has been established in similar population based studies where 1-7% has been infected [21-25,31]. It is however in the lower range of earlier published studies from Norway, partly because these reflect a population who has been seeking health care [29,32-34]. The proportion positive in the intervention group should be interpreted with some caution due to potential selection bias. Those who believe they are infected are probably more likely to get tested, also with home sampling.

### Secondary outcome – proportion treated among the diagnosed

We observed a small, although not significant, difference in proportion treated among the diagnosed in the two groups. Possible explanations for this observation are that those who seek the health care system for chlamydia testing have a more active health seeking behaviour. Thus, they are more likely to take the treatment prescribed. It may also be an additional obstacle to seek the doctor for a prescription after having received the test result by post. However, a bigger difference in the treatment gap was observed between men and women than between the two treatment groups suggesting that women have a more active health seeking behaviour than men.

This study was a comparison between usual care and a home based screening intervention. Other screening approaches such as using general practise for pro-active and systematic screening have shown high testing rates in other studies [35,36]. Assessing such approaches was outside the scope of this study.

### Limitations

The study design did not allow blinding of the assigned study regimen to the participants. With such a large intervention affecting a fourth of the population in the targeted age group, there is bound to have been some “leakage” of information to the control group. Thus, also control subjects may have been prompted to get a chlamydia test. This effect would lead to a lower estimate of the effect of the intervention.

It was not possible to blind the laboratory personnel because home tests from the intervention group were the only home tests received at this laboratory. However, all tests received were analysed according to the normal procedures in the laboratory.

An unknown proportion of our study population lives temporarily somewhere else than their address in the population register. As patients tested for chlamydia in health facilities outside the county could not be assessed, the actual testing rate is likely to be higher in both study groups than what was observed in this study.

The randomization produced unbalance between the groups concerning municipality of residence (in one out of 26 municipalities). This could be a source of bias if the pre-trial prevalence of chlamydia differed between the municipalities. We do not know this. However, adjustment for municipality in the analysis had minimal effects on the risk ratio estimates.

The trial tested only a single round of screening with an observation time of three months for both intervention and control groups. The aim of the intervention was to convince people who were not contemplating going to their physician for chlamydia testing, to get tested early. Because we had only three months follow up for both groups, we cannot exclude that the intervention only brought forward in time testing that would otherwise have occurred later. The same limitation is also seen with Scholes et al. and Andersen et al. [21,25]. However, a single round of screening does not show an effect on community prevalence of chlamydia over time. A recent study from the Netherland assessed the effect of yearly systematic screening after three rounds of screening [37]. The authors conclude that three yearly rounds of register based chlamydia screening did not reduce the chlamydia prevalence when compared with the control population mainly because the screening uptake was too low. In this study the initial uptake was similar to our study but down to 9.5% after the third round [37].

The intervention consisted of both a letter stating the importance of chlamydia testing and a home sampling kit. We cannot differentiate which of these elements that led to the positive effect on testing rates. We cannot exclude that an informative letter alone could have produced a similar effect.

The study did not take into account the economic aspect of the intervention. Cost effectiveness analyses have been carried out on systematic home based screening in other countries. A Danish study concluded that the screening programme saved societal costs and should be considered an alternative to in-office screening [38]. In the Netherlands, cost effectiveness analyses indicated that the screening program, with similar uptake as in our trial, would not lead to acceptable levels of major outcomes averted or adjusted life years gained [39]. In our study, 10 000 home sampling packages by mail led to 1653 individuals tested, 105 diagnosed and 89 treated for chlamydia. The risk difference of treatment between the intervention and control group was 0.54%. This means that 185 people (1/0.54%) would need to be offered the intervention in order to get one more infected persons treated. Further research is therefore needed to evaluate the cost-effectiveness of such an intervention also in the Norwegian context.

This study’s final outcome was treatment for chlamydia. However, the ultimate objective of treatment for chlamydia is to reduce the number of complications and thereby improve reproductive health. Since we have no follow-up for pelvic inflammatory disease (PID) or other complications, we have not shown that screening with home sampling will in fact lead to a reduction of complications. Østergaard et al. showed that a single round of screening with home sampling was associated with a lower proportion of self reported cases of PID after one year compared with a strategy of sampling in the health care system [40]. However after a nine year follow up there was no difference in reproductive complications between the two groups [41]. Oakeshott et al. suggests that the effect of a single round of chlamydia screening in preventing PID may have been overestimated [42]. The effect of repeated screening rounds is unknown.

### Implications

We have shown that screening with home sampling in a Norwegian county increased the number of tested, diagnosed and treated compared with no intervention in the three months following the intervention. The testing uptake in this trial was 16.5%. Given the rather homogenous nature of Norwegian society and health care system, we think that the trial results are generalizable to the rest of Norway and possibly to similar countries. The same initial uptake level was in the Netherlands shown to be insufficient to reduce the chlamydia prevalence, and the uptake dropped additionally in the following two screening rounds [37]. At present home based systematic screening cannot be recommended before more research has established the effect of home sampling and other screening strategies to enhance early diagnosis and treatment on the occurrence of PID and the chlamydia epidemic.

## Conclusion

Systematic screening with information and home sampling increased the number of Norwegian men and women between 18–25 year of age getting tested, diagnosed and treated for chlamydia compared to the current strategy of sampling in the health care system. The intervention led to five times more people getting tested and to 2.5 times more infected people getting treated in the first three months following the intervention. However, this study has not established that the intervention will lead to either reduced chlamydia prevalence nor reduce the incidence of complications caused by chlamydia. Further research is therefore needed to determine the long-term impact of this and other screening strategies before such programmes can be recommended.

## Competing interest

The authors declare that they have no conflict of interest, financial or non-financial. The research was funded by the Norwegian Institute of Public Health.

## Authors’ contributions

HK has planned and designed the research study, been involved in the acquisition of data, done the analysis of and interpretation of data and drafted and revised the manuscript. ON has participated in the design of the research project, made a substantial contribution to the acquisition of data and has been involved in revising the manuscript critically. AaT has made a substantial contribution to the analysis and interpretation of data and has been involved in revising the manuscript critically. PAa has been involved in the planning and design of the research study, participated in the analysis and interpretation of data and has made a substantial contribution to the draft and revision of the manuscript. All authors have read and approved the final manuscript.

## Pre-publication history

The pre-publication history for this paper can be accessed here:

http://www.biomedcentral.com/1471-2334/13/30/prepub
